# Citrate of Caffeine in Hemicrania

**Published:** 1851-02

**Authors:** 


*Citrate of Caffeine in Hemicrania.*—Dr. Hannon advocates very strenuously the use of the above salt in hemicrania. We find in the *Presse Médicale*, of Brussels, two long articles on the subject, with some cases and a good description of the caffeine and its preparation. It is to be given in doses, varying from 5 grains to 1 drachm; and the author has added formulæ for administering the citrate in the shape of pills, draught, syrup, lozenges, &c.—*Ibid.*

